# Improving Theaflavin-3,3′-digallate Production Efficiency Optimization by Transition State Conformation of Polyphenol Oxidase

**DOI:** 10.3390/molecules28093831

**Published:** 2023-04-30

**Authors:** Ying Huang, Changzheng Gao, Wei Song, Wanqing Wei, Xiulai Chen, Cong Gao, Jia Liu, Jing Wu, Liming Liu

**Affiliations:** 1 School of Food Engineering, Anhui Science and Technology University, Chuzhou 233100, China; 2 Department of Cardiology, Affiliated Hospital of Jiangnan University, Wuxi 214122, China; 3 School of Life Sciences and Health Engineering, Jiangnan University, Wuxi 214122, China; 4 State Key Laboratory of Food Science and Technology, Jiangnan University, Wuxi 214122, China

**Keywords:** polyphenol oxidase, protein engineering, transition state, theaflavin-3,3′-digallate

## Abstract

Theaflavins (TFs) are good for health because of their bioactivities. Enzymatic synthesis of TFs has garnered much attention; however, the source and activity of the enzymes needed limit their wide application. In this study, a microbial polyphenol oxidase from *Bacillus megaterium* was screened for the synthesis of theaflavin-3,3′-digallate (TFDG). Based on structural and mechanistic analyses of the enzyme, the O-O bond dissociation was identified as the rate-determining step. To address this issue, a transition state (TS) conformation optimization strategy was adopted to stabilize the spatial conformation of the O-O bond dissociation, which improved the catalytic efficiency of tyrosinase. Under the optimum transformation conditions of pH 4.0, temperature 25 °C, (−)-epigallocatechin gallate/epicatechin gallate molar ratio of 2:1, and time of 30 min, Mu_4_ (*Bm*Tyr^V218A/R209S^) produced 960.36 mg/L TFDG with a 44.22% conversion rate, which was 6.35-fold higher than that of the wild type. Thus, the method established has great potential in the synthesis of TFDG and other TFs.

## 1. Introduction

Theaflavin (TFs) is a flavanol with a benzotoketone ring system produced by the condensation of gallocatechin and catechin [1]. TFs have various health benefits, including antioxidant, antiobesity, anti-inflammatory, and anticancer properties, and they can also prevent fatty liver and cardiovascular diseases [2,3,4,5,6]. TFs are naturally derived from the enzymatic oxidation of catechin monomers in fresh green tea leaves during the fermentation process. In addition to the four main compounds, namely theaflavin (TF), theaflavin-3,3′-digallate (TFDG), theaflavin-3′-gallate (TF-3′-G), and theaflavin-3-gallate (TF-3-G), more than 30 species of theaflavins have been found [7]. Among these, TFDG is generally regarded as the most effective bioactive component [8].

The production of TFDG involves direct extraction, chemical oxidation, or enzymatic methods [9]. However, the direct extraction is not suitable for industrial-scale production because of high costs, wastage of resources, and a low extraction rate. The chemical oxidation synthesis method has disadvantages such as a low extraction rate of TFs and a complicated operational process. Furthermore, according to the European and United States legislation, chemically synthesized additives cannot be used in cosmetics, foods, or beverages [10]. Consequently, the enzymatic synthesis of TFDG has garnered increasing attention, which is a natural synthesis method and can greatly decrease the production cost [11].

Polyphenol oxidase (PPO) and peroxidases (PODs) are two groups of enzymes that are important for the synthesis of TFs. Zhang et al. [12] integrated transcriptome and metabolite analysis of tea leaves to identify PPO and POD genes that may be involved in TF production during tea processing. The results showed that PPOs mainly catalyze the oxidation of catechins to TFs, whereas PODs catalyze the oxidation of catechins to TFs and further catalyze the rapid polymerization of TFs to thearubigins. Therefore, PPO are the key enzymes in TF synthesis. To synthesize TFDG, purified PPOs from tea leaves were used as catalysts, which obtained a final yield of 11 mg/L at pH 5.5 and 33 °C [13]. In a different study, PPOs taken from pear fruits were purified, immobilized, and applied to the synthesis of TFDG, using (−)-epigallocatechin gallate (EGCG) and epicatechin gallate (ECG) as substrates, and resulted in a maximum yield of 42.23% [11]. These previous studies have made remarkable progress in the development and utilization of plant PPO. However, the process of isolating and purifying PPO directly from plant tissues is complex and inefficient. Therefore, identifying new and efficient PPO for TF production is important.

Computational strategies have emerged in recent years that become an effective and cost-effective way to obtain target mutants by virtual screening. Rational design is based on structural analysis and applies various computational strategies to design novel enzymes or to optimize protein properties using a limited number of mutations [14,15]. This approach has achieved remarkable success in industrial biology and synthetic biology. The transition state (TS) is a metastable state during a chemical reaction that bridges reactants and products. Enzymatic catalysis and TS theory indicate that the catalytic effect of enzymes is owed to the TS, which binds to the enzyme much more tightly than the substrate [16]. Based on this TS theory, much progress has been made in de novo enzyme design and protein modification. Therefore, the use of simple and efficient computational methods is highly preferable for enzyme engineering. Combining the advantages of directed evolution and rational design, we used a method of enzyme redesign based on the Rosetta enzyme design for the computer simulation of stable TS in order to improve oxidation efficiency.

In this study, a microbial PPO named *Bm*Tyr was screened. Based on the analysis of its structure and mechanism, the O-O bond dissociation (TS2) was found to be the rate-determining step. Therefore, we proposed a strategy to optimize the conformation of TS2, based on the transition state and structure. Compared with the traditional approach of random mutation, which required a lot of screening work, this strategy effectively reduced the workload. The best variant was *Bm*Tyr^V218A/R209S^, which increased the specific activity by 4.67-fold and V_max_/*K*_m_ by 6.97-fold toward EGCG. Finally, the optimization of the transformation conditions obtained a titer of 960.36 mg/L TFDG.

## 2. Results

### 2.1. Screening Enzymes for TFDG Synthesis

**Figure 1:**
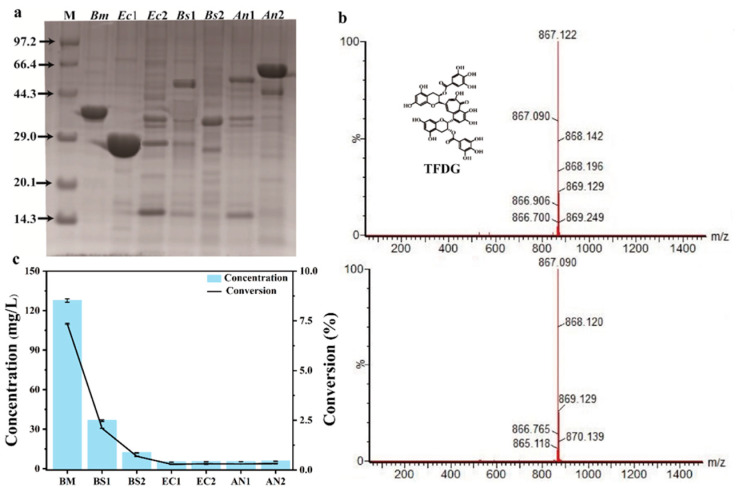
(**a**) SDS-PAGE analysis of the purified protein (M, Maker; *Bm*, *Bacillus megaterium* ACC86108.1, *Ec*1, *Escherichia coli 8739* AYB96806.1, *Ec*2, *Escherichia coli* AAC75642.1, *Bs*1, *Bacillus subtilis* MG571271.1, *Bs*2, *Bacillus subtilis strain N2* KY849863.1, *An*1, *Aspergillus niger ATCC 1015* EHA19536.1, *An*2, *Aspergillus niger ATCC 13496*) CAK40046.1. (**b**) Characterization of TFDG with HPLC-MS. (**c**) Effect of different activities of seven recombinant PPOs (5 mM substrate in 200 mM Mcilvaine Buffer (pH 5.0), 30 °C, 0.1 mM Cu^2+^) on TFDG production. The data represent mean ± s.d., as determined from three independent experiments.

PPO from three categories including tyrosinases, catechol oxidases, and laccases, which could produce quinone, were selected as probes on the basis of the specified product query tool in the BRENDA Enzyme database. Using these probes, seven functionally known microbial PPOs from the NCBI database were selected through data mining. The selected PPOs were subcloned into the pET28a (+) vector and were heterologously expressed in *E. coli* BL21 (DE3). SDS–PAGE analyses were performed to observe soluble PPO proteins (Figure 1a, which were then purified to produce TFDG). Among them, the enzyme *Bm*Tyr from *Bacillus megaterium* showed the highest yield (127.62 mg/L TFDG) (Figure 1c). The product was identified by HPLC (Figure S1), and mass spectroscopy showed the [M−H]^−^ ion at m/z 867, which was consistent with the standard sample (Figure 1b). Therefore, *Bm*Tyr was selected as a catalyst for TFDG production. However, the conversion rate was only 7.35%. Hence, improved *Bm*Tyr catalytic efficiency is required. To guide the engineering-improved *Bm*Tyr, the structure and mechanism of *Bm*Tyr were determined.

### 2.2. Structure and Catalytic Mechanism of BmTyr

**Figure 2:**
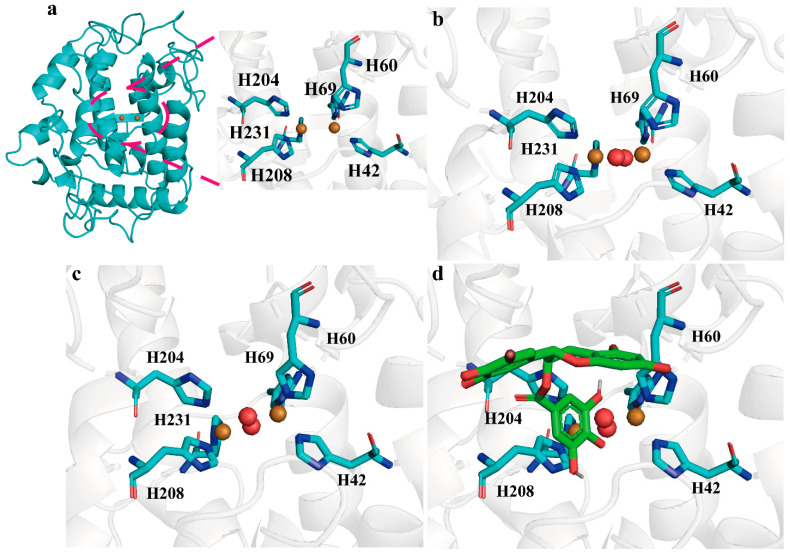
The construction process of the theoretical complex structure. (**a**) The whole structure and detailed active site view of 3nm8. (**b**) The detailed active site view of M_1_. (**c**) The detailed active site view of M_2_. (**d**)The detailed active site view of M_3_. The carbon of histidines is shown in cyan stick and Cu^2+^ in brown sphere.

*Bm*Tyr structure was obtained from the PDB database (PDB:3nm8) (Figure 2a), which was similar to that of the type 3 copper protein family containing two Cu (II) ions. These two Cu(II) ions were coordinated by six conserved histidine residues, which serve as the major cofactors. We aimed to identify the complex structure of *Bm*Tyr with Cu (II) ions and a ligand (EGCG); however, only the *apo-Bm*Tyr structure was obtained with 2.56 Å resolution (PDB ID:8HPI) (Figure S2). Therefore, based on 3nm8, a reactive binding mode M1 was created (Figure 2b). M1 contained two copper ions that were 3.5 Å apart and two bridging solvent molecules that were designated as oxygens. Furthermore, an MD analysis of M1 was performed, and the conformation with the highest evaluation rate was obtained through cluster analysis (Figure 2c). This conformation was named model 2 (M2), which was used for subsequent docking experiments with EGCG. The docking experiments produced a theoretically complex structure of M2–EGCG, which was named M3 (Figure 2d and Figure S3).

**Figure 3:**
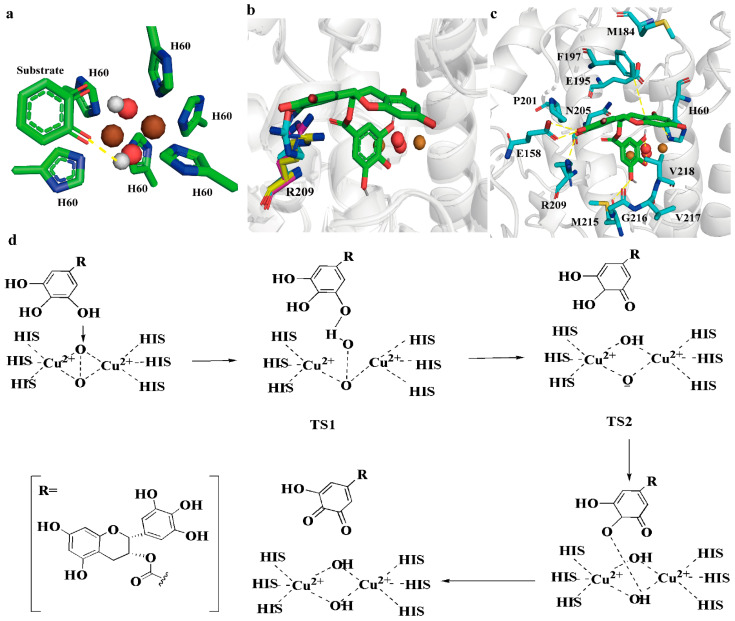
EGCG binding and catalytic mechanism of *Bm*Tyr. (**a**) Possible 3D view structure for TS3. (**b**) The three conformations of R209 identified in the molecular dynamics are presented. (**c**) The binding of EGCG in the active site of *Bm*Tyr. The carbon of EGCG is shown in green stick and the remaining residues are shown in cyan. (**d**) Catalytic mechanism of *Bm*Tyr.

The docking results are shown in Figure 3a. The flexibility of R209 facilitated the stabilization of EGCG in the *Bm*Tyr binding pocket. F197, a bulky residue, showed a weak π–π interaction with the aromatic ring of EGCG, forming a specific gate that prevented a substrate with incorrect orientation from entering the pocket (Figure 3b). The conserved water molecules, which were stabilized by E195 and N205, acted as proton donors and receptors during the process of hydroxylation. The other residues, including M184, P201, G216, V217, and V218, interacted to fix EGCG to facilitate the reaction (Figure 3b).

Based on the docking results and the reported mechanism underlying PPO-mediated reactions, a hypothetical catalytic mechanism of *Bm*Tyr was inferred (Figure 3c) [17,18,19,20]. This reaction contained the following steps: (i) phenolic -O11H bond formation with the bridging oxygen atom (TS1), (ii) O-O bond dissociation of the bridging oxygen species (TS2), (iii) another phenolic -O9H bond formation, and (iv) proton transfer from the catechol to the bridging oxygen group accompanied by electron transfer to afford the quinone product. A detailed analysis of the energy profile showed that the rate determination step is the O-O bond dissociation, regardless of the active copper center state [21]. Similar to the catechol oxidase diphenolase cycle, three protons are required to enter the stage where the O-O bond is cleaved [22]. The O-O bond cleavage was a prohibitively large barrier for this reaction. MD simulations showed that the distance between the -O11H of the EGCG and the O1 of the bridging oxygen atom of the active center was 4.66 ± 0.57 Å (Figure S8), which made the hydride transfer and proton transfer difficult. Thus, optimizing this critical step required the optimum conformation of TS2.

### 2.3. Improving BmTyr Catalytic Efficiency via TS2 Conformation Optimization

**Figure 4:**
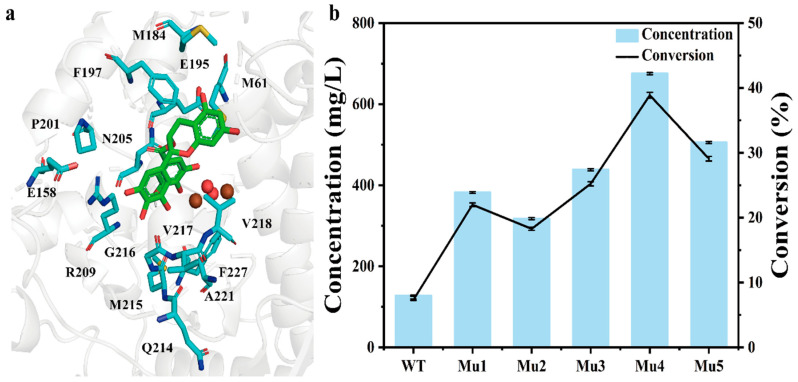
Redesign of *Bm*Tyr. (**a**) Residues (cyan sticks) within 5 Å from the substrate (green sticks) in the *Bm*Tyr. (**b**) The activity of the designed enzymes on TFDG production (5 mM substrate in 200 mM Mcilvaine Buffer pH 5.0, 30 °C, 0.1 mM Cu^2+^). The data represent mean ± s.d., as determined from three independent experiments.

To determine the optimum conformation of TS2, restrained MD simulations were conducted. First, the MD state model was constructed by defining geometric criteria on the basis of the existing M3 and previous studies on the mechanism [20]. During the restrained MD calculations, the coordinates of the active site atoms were fixed. Subsequently, stable conformations were selected by cluster analysis to construct the active site cluster model, namely the TS2 state model (Figure S5). A total of 15 residues (M61, E158, M184, E195, F197, P201, N205, R209, Q214, M215, G216, V217, V218, A221, and F227) within 5 Å of the TS2 were selected for the subsequent Rosetta enzyme design (Figure 4a).

During Rosetta enzyme design, to accommodate the large backbone changes introduced at each step, we used PSSMs to limit amino acid choices to identities that were common in the multiple-sequence alignment (Figure S6) [23]. Thus, 119 mutants were designed in this study. Computational mutation scanning was performed to predict the effects of each mutation (ΔΔG). Mutants with scores lower than that of the WT at the same residue position were selected as hits, which created 22 variants for experimental validation (Figure S7). Using PSSMs and ΔΔG ≤ 0 R.e.u. as the cutoff, severely destabilizing mutations were eliminated.

The 22 variants were expressed in *E. coli* BL21 (DE3), which were purified and tested for TFDG production (Figure S7). Compared with the TFDG titers of WT, 19 of the 22 variants showed improved TFDG titers, which confirmed the effectiveness of this method. Among the 19 improved variants, Mu_1_ (*Bm*Tyr^R209S^), Mu_2_ (*Bm*Tyr^V217L^), and Mu_3_ (*Bm*Tyr^V218A^) showed an almost two-fold increase in TFDG titer; Mu_3_, the most efficient mutant, showed a 2.43-fold increase in TFDG titer (438.19 mg/L) compared with that of WT. To further increase this activity, R209S and V217L were combined with V218A, resulting in the variants Mu_4_ (*Bm*Tyr^V218A/R209S^) and Mu_5_ (*Bm*Tyr^V218A/V217L^). Mu_4_ showed the highest TFDG titer (675.30 mg/L), corresponding to a 4.29-fold increase compared with that of the WT (Figure 4b). The kinetic parameters of WT and its variants are shown in Table 1. Compared with WT, Mu_4_ showed increased activity toward ECG and EGCG. The specific activities of Mu_4_ toward ECG and EGCG, which were 51.14 and 86.58 U/mg, respectively, were 3.68- and 4.67-fold higher than that of the WT. The V_max_/*K*_m_ of Mu_4_ toward ECG and EGCG were 14.59 U and 27.17 U min^−1^ mg^−1^ mM^−1^, respectively, which were 6.05- and 6.97-fold higher than that of the WT.

**Figure 5:**
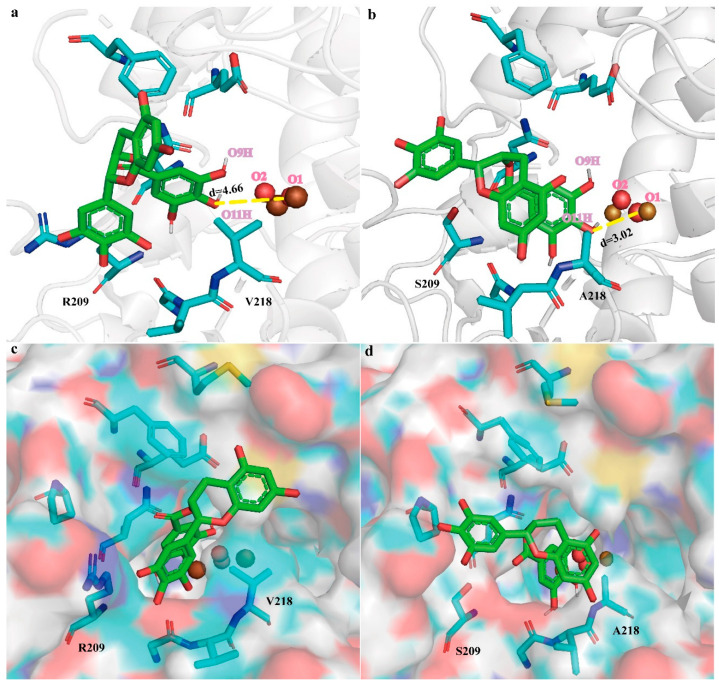
MD simulations on the *Bm*Tyr-EGCG and Mu_4_-EGCG complexes. Representative MD snapshots of the *Bm*Tyr-EGCG (**a**) and Mu_4_-EGCG (**b**) complexes. Binding site of WT with bound EGCG (**c**) and Mu_4_ with EGCG (**d**). Residues are shown in the cartoon and surface, respectively. The carbon of EGCG is shown as a green stick, and the remaining residues are shown in cyan.

To elucidate the mechanism underlying the increased enzymatic activity, MD simulations were performed on the Mu_4_–EGCG complex. Compared with *Bm*Tyr–EGCG, the hydride transfer distance of the Mu_4_–EGCG complex was shortened from 4.66 ± 0.57 to 3.02 ± 0.31 Å (Figure 5a,b and Figure S8). This suggests that the distance between the -O11H of the EGCG and the O1 of the bridging oxygen atom of the active center was shortened in Mu_4_. Furthermore, V218A and R209S eliminated steric hindrance (Figure 5c,d). The side chain residues of valine and arginine in Mu_4_ were larger than that of the WT, which increased the steric hindrance of the transition state. Providing more space for the substrate, where the catalytic residues are present, is conducive to the stability of the transition state, which may enable the release of the products.

### 2.4. Optimize the Transformation System to Produce TFDG

**Figure 6:**
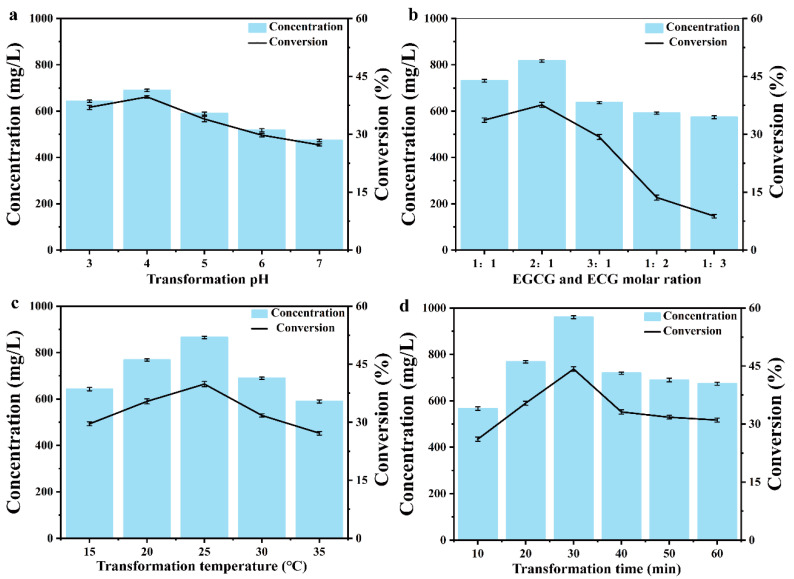
Optimization of TFDG production conditions. (**a**) Effect of buffer pH on TFDG production. (**b**) Effect of EGCG and ECG ration on TFDG production. (**c**) Effect of temperature on TFDG production. (**d**) Effect of time on TFDG production. This system contains 200 mM MacIlvaine buffer, 0.1 mM Cu^2+^ and 0.06 mM pure enzymes with 200 rpm. The data represent mean ± s.d., as determined from three independent experiments.

Purified Mu_4_ was used as the catalyst to produce TFDG. To increase the TFDG titer by Mu_4_, the reaction parameters that affect TFDG synthesis were optimized (Figure 6a,d), including pH (3.0–7.0), EGCG/ECG molar ratio (1:1, 1:2, 1:3, 2:1, and 3:1), reaction temperature (15–35 °C), and reaction time (10–60 min). The optimum conditions for the efficient synthesis of TFDG by Mu_4_ were as follows: Mcilvaine Buffer pH 4.0, 2:1 molar ratio of EGCG/ECG, temperature at 25 °C, and a reaction time of 30 min. The highest TFDG titer was 960.36 mg/L in 30 min, which was 6.35-fold higher than that of WT (127.62 mg/L).

## 3. Discussion and Conclusions

In this study, the efficacy of PPO from different sources in TFDG synthesis was compared, and *Bm*Tyr, with a high conversion rate and wide application value, was screened. To improve the conversion of TFDG, we performed mechanism analysis and computational design to optimize the TS2 conformation. TFDG was effectively synthesized using ECG and EGCG as substrates and the optimum mutants as biocatalysts, increasing the conversion of TFDG by 6.35-fold. Therefore, the established method has practical value for TFDG synthesis and great potential for the synthesis of other TFs.

A stable enzyme source-supporting method, using microbiological fermentation, was selected. To date, most enzyme sources related to in vitro catalytic synthesis of TFDG were derived from plant extracts, which are mainly concentrated in natural PPOs that are directly isolated from tea leaves, mushrooms, or pear fruits [13,24]. Plant PPOs are not conducive to the industrialization of tea pigment production because it requires a large number of tea leaves to meet the application requirements. Furthermore, there were large differences in the enzyme composition of plants in different seasons, and their effects on the synthesis of TFs were inconsistent. The large-scale production of TFs requires a stable enzyme source. We selected seven functionally known microbial PPOs from the NCBI database via data mining. The recombinant strain *Bm*Tyr was selected to improve the biological preparation of TFDG because it has the advantages of a simple culture method, abundant raw material sources, high enzyme yield, and good enzyme production stability.

A transition-state conformation optimization strategy was adopted, which provided an effective tool for obtaining beneficial mutations in *Bm*Tyr to improve TF synthesis. Many strategies have been adopted to engineer proteins and to improve their properties. For example, Zhou et al. [25] obtained activating mutations from *Bm*Tyr after screening almost 20,000 colonies for random mutations. This approach required enormous screening efforts. In this study, *Bm*Tyr was efficiently engineered on the basis of the Rosetta enzyme design for the computer simulation of stable TS to improve oxidation efficiency. A total of 19 beneficial mutants were obtained from the designed library (22 mutants) that produced TFDG. Compared with the traditional random mutation, this strategy effectively reduces the workload. In a different study, theaflavins were synthesized by extracting polyphenol oxidase from eggplants. The results showed that the total content of theaflavins could reach 40.7 mg under optimum reaction conditions [26]. Ngure, F. M. et al. [27] used PPO from tea to oxidize catechins, and the final theaflavin content reached 26.99 µmol/g. PPOs from snow lotus fruits have also been used to oxidize catechins to form theaflavins. The final estimate of theaflavins in the reaction system was 65 μM dry weight/h [28]. Under the catalysis of the optimum mutant *Bm*Tyr^V218A/R209S^, the titer of TFDG reached 960.36 mg/L with 44.22% conversion, which was 6.35-fold higher than that of the WT. These results showed the effectiveness of our strategy and may be useful for improving the properties of other enzymes.

Despite the abovementioned results, several factors restrict the production of TFs. Although the TFDG generation rate is high, the industrial application of free enzymes is impeded because they are either inactivated or cannot be recovered for reuse. Immobilized enzymes can effectively overcome the limitations of free enzymes. A suitable carrier should be found to immobilize PPO, which can make the enzyme reaction continuous and automatic, reduce production costs, and improve efficiency. Our ongoing study will address these issues and improve the utilization of *Bm*Tyr.

## 4. Materials and Methods

### 4.1. Strains, Plasmids, and Chemicals

All plasmids and strains are listed in Table S1, and their genetic constructions are shown in the supporting information. Commercial solvents, standards, and reagents were purchased from Meryer Chemicals (Shanghai, China), Sigma Aldrich (Shanghai, China), J&K Chemicals (Beijing, China), TCI Chemicals (Shanghai, China), and Aladdin Reagents (Shanghai, China) and used without further purification.

### 4.2. Access Codes

Biosynthetic protein accession codes: *Bm*, ACC86108.1, *Ec*1, AYB96806.1, *Ec*2, AAC75642.1, *Bs*1, MG571271.1, *Bs*2, KY849863.1, *An*1, EHA19536.1, *An*2, CAK40046.1.

### 4.3. Culture and Purification

All desired purified proteins and mutants were obtained under the following operating conditions. Strains were first grown in LB medium with suitable antibiotics at 37 °C and 200 rpm. When the OD_600_ of the culture broth reached 0.6–0.8, IPTG was added to make a final concentration of 0.1 mg/mL. The cells were inducted at 16 °C for 16 h, collected by centrifugation (5000 rpm, 10 min), suspended in lysis buffer (20 mM Tris-HCl (pH 8.0), 20 mM imidazole, 500 mM NaCl) and then disrupted using a high-pressure homogenizer. The resulting crude lysate was centrifuged at 10,000 rpm at 4 °C for 30 min, and the protein containing a His-tag was trapped on Ni-NTA Superflow resin for 30 min. Subsequently, the elution buffer (20 mM Tris-HCl (pH 8.0), 0.3 M NaCl, 0.5 M imidazole) was used to release the protein. The protein concentration was determined using a NanoDrop 2000c spectrophotometer (Thermo Scientific). The purity of the proteins was determined by SDS-PAGE.

### 4.4. Construction and Screening

The seven samples of PPOs obtained by genome mining were overexpressed in *E. coli* via pET28a and purified to obtain pure enzymes that were used to catalyze the conversion of ECG and EGCG to TFDG. This 2 mL screening system contained 0.2 M MacIlvaine buffer, 5 mM substrate, 0.1 mM Cu^2+^, and 0.06 mM pure enzymes at pH 5.0, 30 °C, and 200 rpm.

### 4.5. HPLC Analysis

The titer of TFDG was determined using high-performance liquid chromatography (HPLC). The HPLC analytical conditions were as follows: XBridge^®^-C18 column (5 μm, 4.6 × 250 mm), column temperature of 30 °C, detection wavelength of 280 nm, injection quantity of 10 μL, and the flow rate was 1.0 mL/min. Chromatography was performed with an elution of 30% A for 5 min, 34% A for 3 min, and finally, 30% A for 2 min, where mobile phase A was 100% acetonitrile and mobile phase B was 2% acetic acid aqueous solution.

The conversion was calculated as follows:

Conversion of TFDG (%) = (a/b) × 100

where a is the amount of TFDG synthesized, and b is the amount of ECG added to the system. The conversion values of the reaction catalyzed for TFDG synthesis were calculated.

### 4.6. Protein Crystallization and Structure Determination

Crystals of apo-*Bm*Tyr were obtained after 3 days at 20 °C in 96-well plates, in which 0.8 μL protein solution (25 mg/mL) and an equal volume of reservoir solution (0.1 M zinc acetate dehydrate, 0.1 M sodium acetate trihydrate; pH 4.6; 12% *w*/*v* PEG4000) were mixed, as per the optimized conditions for crystallization. All crystals were flash frozen with 25% glycerol as an antifreezing solution in liquid nitrogen. X-ray diffraction data were collected using a Bruker D8 QUEST diffractometer, and the data sets were indexed, integrated, and merged using XDS [29]. The structure of *Bm*Tyr was solved using the molecular replacement, with the protein structure of 3nm8 as the search model. Iterative cycles of model building and refinement were performed in Coot [30] and Refmac [31] programs. The data collection and refinement statistics of the apo-*Bm*Tyr crystal structure are listed in Table S4. Structural figures were prepared using PyMOL v2.3.3 (Schrödinger, LLC, New York, NY, USA).

### 4.7. Initial Structural Preparation

The initial structure of *Bm*Tyr was obtained using *Bacillus megaterium* as a template (PDB ID:3nm8 [32]). Based on 3nm8, a reactive binding mode, namely *Bm*Tyr (M1), was created. The protonation states of the charged residues were determined at a constant pH of 5.0 on the basis of the pKa calculations using H^++^ [33] while taking into account the local hydrogen bonding network. The His residues were assigned as HID. Asp and Glu residues were deprotonated, whereas Lys and Arg were protonated. The prepared protein was neutralized by adding Na^+^ ions and solvated into a truncated octahedral TIP3P [34] water box with a 10 Å buffer distance on each side. The M1 system consisted of 27,473 atoms. The system was equilibrated through a series of minimization steps that was interspersed by short molecular dynamics (MD) simulations, during which the restraints on the protein backbone heavy atoms were released gradually (with a force constant of 10, 2, 0.1, and 0 kcal/mol·Å^2^). The system was gradually heated up to 303 K in 50 ps in which harmonic potentials were used to positionally restrain the protein backbone heavy atoms (with a force constant of 10 kcal/mol·Å^2^). Finally, the standard unrestrained MD simulation with periodic boundary condition at 303 K and 1 atm was carried out for up to 100 ns. Based on the dynamically stable *Bm*Tyr (M2) binary complex structure determined by MD simulations, EGCG was docked into the active center to obtain the possible binding mode of EGCG (*Bm*Tyr–EGCG, M3). A representative pre-equilibrated structure of *Bm*Ty–EGCG was used as a template to create the starting coordinates of Mu_4_–EGCG by changing the two mutated residues (V218A/R209S). To check for statistical convergence, each simulation was carried out in triplicate with different initial atomic velocities that were randomly generated according to the Maxwell–Boltzmann distribution.

### 4.8. Molecular Docking

In the equilibrium trajectories of the M2 binary complex, 2000 snapshots were selected and divided into ten groups through a hierarchical agglomerative (bottom-up) approach [35]. EGCG was fully optimized at the B3LYP/6-31G(d) level using the Gaussian 16 [36] package, and docked into the active site of each group representative snapshot. Molecular docking was performed by the Lamarckian genetic algorithm local search method in AutoDock 4.2 and AutoDockTools-1.5.6 [37]. The docking approach was employed on a rigid receptor conformation. One-hundred independent docking runs were undertaken. Reasonable conformations were then selected as the binding conformations for MD simulations.

### 4.9. MD Simulations

The pre-equilibrated M3 structures and possible catalytically reactive binding modes of EGCG were used as the starting conformations for the MD simulations of the protein–ligand complexes. The partial charges of EGCG were fitted with HF/6-31G(d) calculations, and the restrained electrostatic potential (RESP) [38,39] protocol was implemented by the Antechamber module in the Amber 16 package [40]. Each system was initially neutralized with Na^+^ counter ions and solvated with TIP3P [34] water in a truncated octahedron box at a 10 Å buffer distance. The resulting systems M2, M3, and Mu_4_–EGCG contained 27,473, 27,188, and 27,230 atoms, respectively. Next, each system was equilibrated with a series of minimization that was interspersed by short MD simulations, during which the restraints on the protein backbone heavy atoms were released gradually (with force constants of 10, 2, 0.1, and 0 kcal/mol·Å^2^). The system was then gradually heated from 0 to 303 K for 50 ps, in which a 10 kcal/(mol·Å^2^) restraint was applied to heavy atoms on the protein backbone. Finally, an extensive MD simulation of 50 ns was performed at constant temperature and pressure. The pressure was maintained at 1 atm and coupled with isotropic position scaling. The temperature was controlled at 303 K using the Berendsen thermostat method [41]. Long-range electrostatic interactions were treated using the particle mesh Ewald (PME) method [42], and a 12 Å cutoff was applied to both PME and van der Waals (vdW) interactions. A time step of 2 fs was employed along with the SHAKE algorithm [43] for hydrogen, and periodic boundary conditions were used. The atomic positions were stored every 2 ps for further analysis. Each system was checked for stability (structure, energy, and temperature fluctuations) and convergence (root mean square deviations (RMSD) of the structures).

### 4.10. Rosetta Design

Active-site positions were selected for design, and at each position, position-specific scoring matrices (PSSMs) were used to constrain amino acid choices to identities. We generated a sequence alignment, and the less favorable amino acids were removed by natural selection. We then used Rosetta computational mutation scanning. Each “allowed” mutation was modeled in the context of the WT structure, and the energy difference (ΔΔG) between the WT and the mutant was calculated. We defined the space of “conformation optimization” mutations as mutations with ΔΔG ≤ 0 Rosetta energy units (R.e.u.), generating many designs for experimental testing.

### 4.11. Directed Evolution Experiments

Directed evolution experiments were performed by whole-plasmid polymerase chain reaction (PCR) using KOD-Plus-Neo (TOYOBO). The primers used are listed in Table S2. The resulting PCR products were digested with D*pn*I to remove the template plasmid. Furthermore, 10 μL of digested products was transformed into *E. coli* BL21 (DE3) cells for subsequent screening or DNA sequencing (GENEWIZ, China). These mutants were cultured into LB medium and shaken at 37 °C for 12 h. Mutants were then diluted 1:10 into 150 mL fresh Terrific Broth (TB) medium. After shaking at 37 °C for 3 h (for cell growth), the temperature was decreased to 25 °C for 16 h (for protein expression). Cells were then collected by centrifugation.

### 4.12. Activity Assay

The activities of *Bm*Tyr and the mutants were spectrophotometrically measured using ECG and EGCG as substrates [44,45]. The activity was determined by measuring the initial rate of quinone formation by an enzyme-labeled instrument under the following conditions: 5 mM substrate, 0.6 mM enzymatic solution containing 0.1 mM Cu^2+^, 200 mM Mcilvaine Buffer (pH 5.0) in a final volume of 200 μL, at 200 rpm and 30 °C for 5 min. Finally, the reaction was terminated by adding 50 μL of 2 M trichloroacetic acid and centrifuging the solution at 12,000× *g* for 5 min. The absorbance of the samples was analyzed at 405 nm, and a reaction mixture without the enzymatic solution was used as a negative control. One unit of enzymatic activity was defined as the amount of enzyme that changes the absorbance by 0.01/min.

### 4.13. Kinetic Assay

The kinetic parameters of the enzymes were determined by measuring the initial rates of product formation at different concentrations of the substrate (EGCG:0–16 g/L, and ECG:0–8 g/L) for 5 min. Other assay conditions were the same as those described for the corresponding activity assays. The samples were withdrawn, extracted, and analyzed using HPLC. The *K*_m_ and V_max_ values were calculated using nonlinear regression according to the Michaelis–Menten equation in the Origin software.

### 4.14. Optimum Reaction Conditions for TFDG Synthesis

The selection of optimal conditions is crucial for the conversion of TFDG. Therefore, the effects of different pH values (pH 3.0, 4.0, 5.0, 6.0, and 7.0), EGCG/ECG molar ratios (1:1, 1:2, 1:3, 2:1, and 3:1, based on a 2.5 mM substrate ECG), reaction temperatures (15, 20, 25, 30, 35 °C), and reaction times (10, 20, 30, 40, 50, and 60 min) on TFDG synthesis were investigated. Optimum parameters were screened by analyzing the effect of each factor on TF production.

## Figures and Tables

**Table 1:** Kinetic constants of purified *Bm*Tyr and its variants.

Enzyme	Substrate	Specific Activity (U mg^−1^)	V_max_ (U min^−1^ mg^−1^ )	*K*_m_ (mM)	V_max_/*K*_m_ (U min^−1^ mg^−1^ mM^−1^ )	E_0_ (J/mol k)
WT	ECG	10.93 ± 0.82	123.24 ± 1.19	59.62 ± 0.91	2.07 ± 0.05	1.28 × 10^−5^
	EGCG	15.26 ± 0.78	35.26 ± 1.25	10.35 ± 0.36	3.41 ± 0.03	1.25 × 10^−5^
Mu_1_ (R209S)	ECG	16.57 ± 0.96	146.26 ± 1.53	43.87 ± 0.12	3.33 ± 0.83	1.29 × 10^−5^
	EGCG	28.63 ± 0.96	116.59 ± 0.95	18.62 ± 0.48	6.26 ± 0.65	1.29 × 10^−5^
Mu_2_ (V217L)	ECG	31.23 ± 0.89	174.53 ± 1.69	28.51 ± 0.48	6.12 ± 0.51	1.29 × 10^−5^
	EGCG	26.04 ± 0.55	145.89 ± 1.04	26.58 ± 0.37	5.48 ± 0.46	1.29 × 10^−5^
Mu_3_ (V218A)	ECG	27.66 ± 0.78	154.69 ± 1.68	36.75 ± 1.27	4.20 ± 0.41	1.29 × 10^−5^
	EGCG	33.78 ± 0.78	165.62 ± 0.98	28.51 ± 0.19	6.51 ± 0.63	1.29 × 10^−5^
Mu_4_ (V218A/R209S)	ECG	51.14 ± 1.05	474.53 ± 10.56	32.51 ± 4.54	14.59 ± 0.64	1.31 × 10^−5^
	EGCG	86.58 ± 0.95	1265.62 ± 13.32	46.58 ± 3.15	27.17 ± 0.88	1.34 × 10^−5^
Mu_5_ (V218A/V217L)	ECG	57.58 ± 1.31	524.69 ± 9.32	33.75 ± 2.56	15.54 ± 0.93	1.32 × 10^−5^
	EGCG	73.16 ± 1.45	865.62 ± 13.46	45.41 ± 4.23	19.06 ± 0.67	1.33 × 10^−5^

The data represent mean ± s.d., as determined from three independent experiments.

## Data Availability

Data are contained within the article and Supplementary Materials.

## Supplementary Materials

The following supporting information can be downloaded at: https://www.mdpi.com/article/10.3390/molecules28093831/s1, Table S1. Strains and plasmids used in this study. Table S2. The primers for gene amplification. Table S3. The primers for the saturated mutation. Table S4. Data collection and refinement statistics. Figure S1. The HPLC chromatograms of TFDG reaction products. Figure S2. The whole structure of *Bm*Tyr (PDB ID:8HPI). Figure S3. The construction process of the whole theoretical complex structure. Figure S4. RMSD and RMSF of *Bm*Tyr^WT^ and *Bm*Tyr^V218A/R209S^. Figure S5. The whole TS2 state model. Figure S6. Multiple sequence alignment of *Bm*Tyr with homologous tyrosinase sequences from superfamily. Figure S7. Redesign of *Bm*Tyr. Figure S8. The distance between the -O11H of the hydroxy oxygen atom of EGCG and the O1 of the bridging oxygen atom of the active center.

## Sample Availability

Compound samples are not available from the authors but available commercially.
