# Supplementary material for: Improving Theaflavin-3,3′-digallate Production Efficiency Optimization by Transition State Conformation of Polyphenol Oxidase
Source: Molecules. 2023 Apr 30;28(9):3831. doi: 10.3390/molecules28093831 (PMC10179947; doi:10.3390/molecules28093831)
Supplement: Supplementary file 1 [file molecules-28-03831-s001.zip › molecules-2317769-supplementary.pdf]

This doc file includes:

Supplementary Table S1 to Table S4

Supplementary Figure S1 to Figure S8

Supplementary materials and methods

Supplementary Tables

Table S1. Strains and plasmids used in this study.

Plasmids or strains	Relevant characteristics
Plasmids	
pET-28a	pBR322 ori, Kan^R , P_{T7} , 6*His, T_{T7}
Strains	
E.coli JM109	For plasmid clone
E. coli BL21(DE3)	For enzyme expression and purification

Table S2. The primers for gene amplification.

Name	Primer (5’ – 3’)
Bm-F	ATGGGTCGCGGATCCGAATTCATGAGTAACAAGTATAGAGTTAGAAAAAACGT
Bm-R	GCAAGCTTGTCGACGGAGCTCTTATGAGGAACGTTTTGATTTTCTTAA
Ec1-F	AGCAAATGGGTCGCGGATCCATGAGTAAGCTGATTGTCCC
Ec1-R	AGCAAATGGGTCGCGGATCCATGAGTAAGCTGATTGTCCC
Ec2-F	CAGCAAATGGGTCGCGGATCCATGAGTAAGCTGATTGTCCCGC
Ec2-R	TTGTCGACGGAGCTCGAATTCTTATATCAGCCAAATGAAACTTGCC
Bs1-F	GTGGACAGCAAATGGGTCGCGGATCCATGAATACATATCACCCATT
Bs1-R	CAAGCTTGTCGACGGAGCTCGAATTCTTATGCCTCCTTCATTCCGA
Bs2-F	CTCGAGTGCGGCCGCAAGCTTTTATTTATGGGGATCAGTTATATCCATC
Bs2-R	CAGCAAATGGGTCGCGGATCCATGACACTTGAAAAATTTGTGGATG
An1-F	ATGGGTCGCGGATCCGAATTCATGCACTTGCATACTATCCT
An1-R	AGTGCGGCCGCAAGCTTTTAGATACCAGAATCATCCTCCTCAA
An2-F	AGCAAATGGGTCGCGGATCCATGACAATCTTTTTGCTACTCCTT
An2-R	TCGAGTGCGGCCGCAAGCTTTTATATACCCGACTCGTAT

Table S3. The primers for the saturated mutation.

Name	Primer (5' – 3')	Name	Primer (5' – 3')
E158D-F	ACCAAAGATGCGCCGACCCTG	V217D-F	GGTCAGATGGGCGATGTGCCG
E158D-R	CGGCGCATCTTTGGTCGCGCC	V217D-R	CGCGGTCGGCACATCGCCCAT
E157Q-F	ACCAAACAGGCGCCGACCCTG	V217L-F	GGTCAGATGGGCCTGGTGCCG
E157Q-R	CGGCGCCTGTTTGGTCGCGCC	V217L-R	CGCGGTCGGCACCAGGCCCAT
F197W-F	CAGCTGGAAGGCTGGATTAAC	V217K-F	GGTCAGATGGGCAAAGTGCCG
F197W-R	CGGGCCGTTAATCCAGCCTTC	V217K-R	CGCGGTCGGCACTTTGCCCAT
P201A-F	TTTATTAACGGCGCGCAGCTG	V217A-F	GGTCAGATGGGCGCGGTGCCG
P201A-R	GTTATGCAGCTGCGCGCCGTT	V217A-R	CGCGGTCGGCACCGCGCCCAT
P201S-F	TTTATTAACGGCAGCCAGCTG	V217R-F	GGTCAGATGGGCCGCGTGCCG
P201S-R	GTTATGCAGCTGGCTGCCGTT	V217R-R	CGCGGTCGGCACGCGGCCCAT
R209A-F	AACCGCGTGCATGCGTGGGTG	V217E-F	GGTCAGATGGGCGAAGTGCCG
R209A-R	ACCGCCCACCCACGCATGCAC	V217E-R	CGCGGTCGGCACTTCGCCCAT
R209N-F	AACCGCGTGCATAACTGGGTG	V218A-F	CAGATGGGCGTGGCGCCGACC
R209N-R	ACCGCCCACCCAGTTATGCAC	V218A-R	CGGCGCGGTCGGCGCCACGCC
R209S-F	AACCGCGTGCATAGCTGGGTG	V218L-F	CAGATGGGCGTGCTGCCGACC
R209S-R	ACCGCCCACCCAGCTATGCAC	V218L-R	CGGCGCGGTCGGCAGCACGCC

Table S4. Data collection and refinement statistics.

**Parameter**	***Apo-BmTyr***
**Unit cell dimensions**	
Space group	P4321
a, b ,c (Å)	111.51, 111.51, 139.66
α, β, γ (Å)	90.00, 90.00, 90.00
Resolution (Å)	46.96-2.30
R_{merge}	0.167
R_{pim}	0.112
I/σI	4.08
Completeness (%)	99.4
No. reflections	39392
R_{work}/R_{free}	0.190/0.237
No. atoms	4998
B factors ($Å^2$)	87.14
bond lengths (Å)	0.0375
bond angles (˚)	1.4670

Supplementary Figures

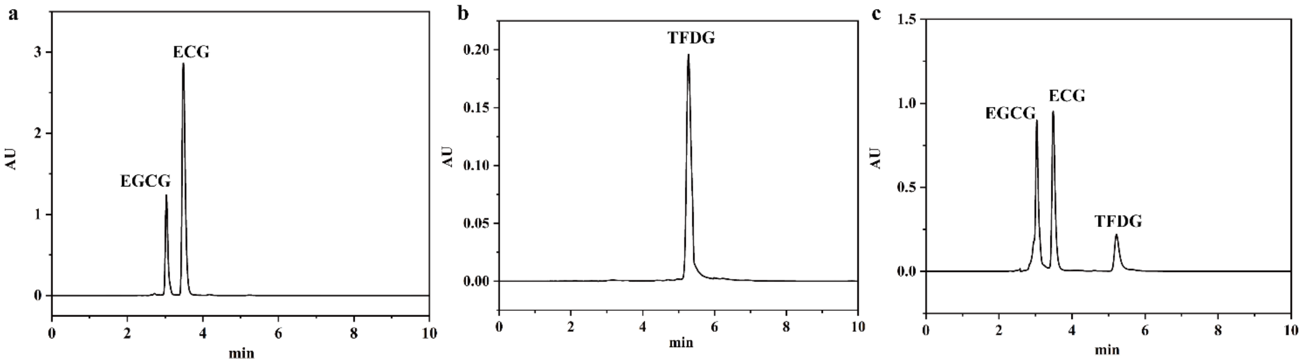

Figure S1. The HPLC chromatograms of TFDG reaction products. (A) The chromatographic peak of ECG, EGCG standard solution; (B) The chromatographic peak of TFDG standard solution; (C) Synthesis of TFDG from ECG and EGCG by *Bm*Tyr.

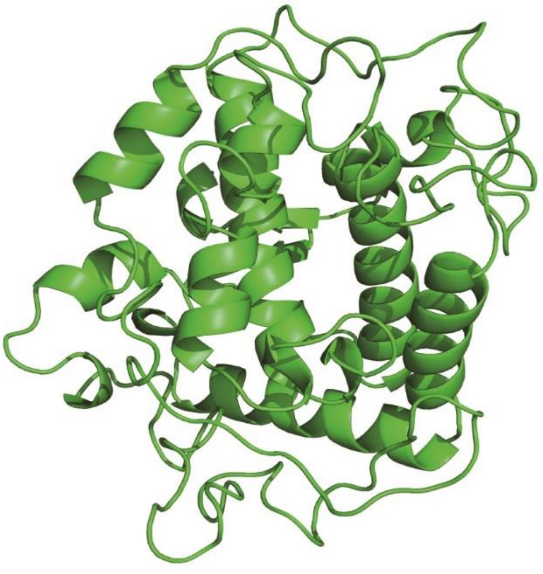

Figure S2. The whole structure of *Bm*Tyr (PDB ID:8HPI).

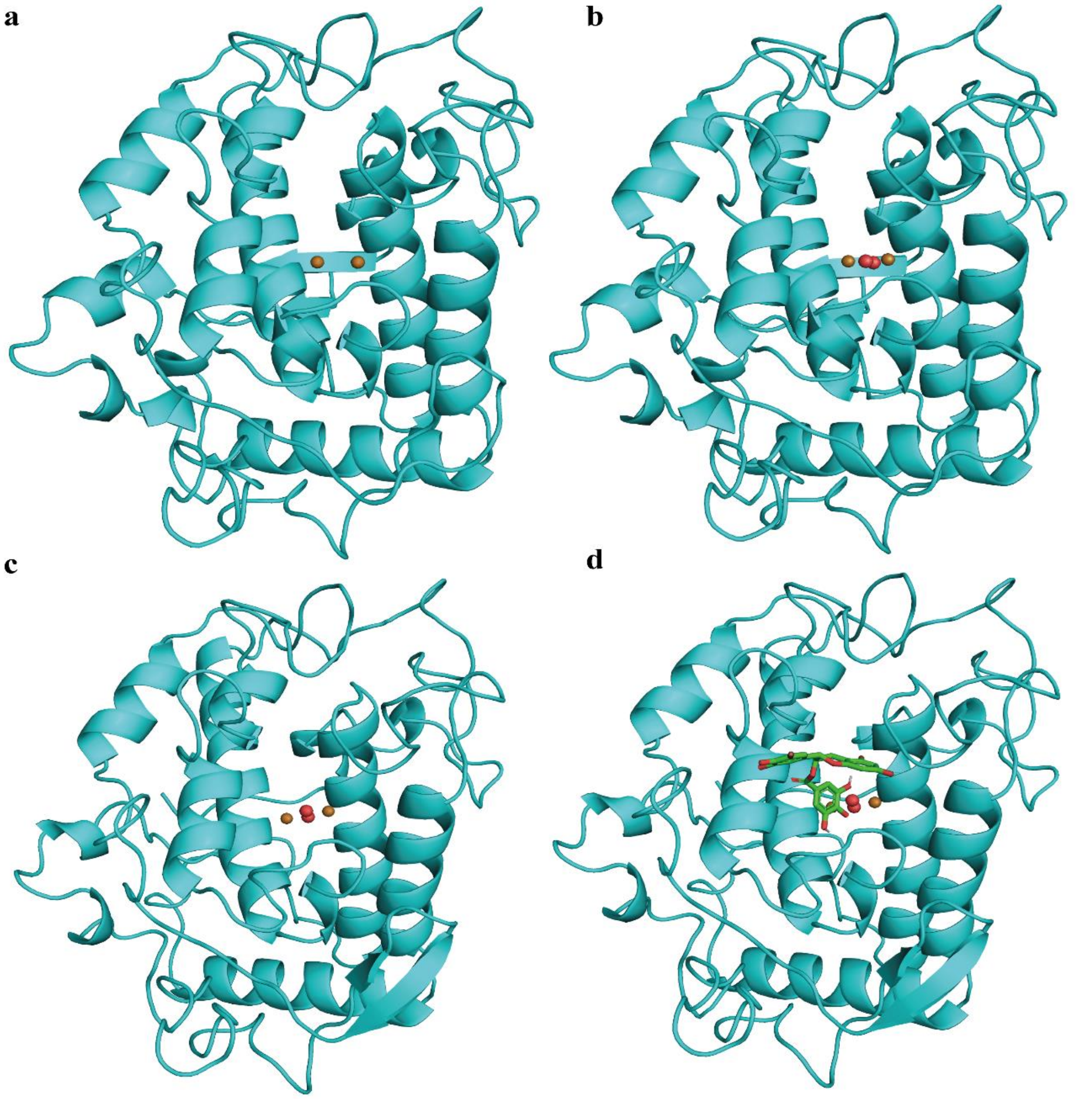

Figure S3. The construction process of the whole theoretical complex structure. (A) The whole structure of 3nm8; (B) The reactive binding mode M1 based on 3nm8; (C) The structure cluster analysis (M2); (D)The theoretical complex structure of BmTyr/Cu^{2+}-EGCG (M3).

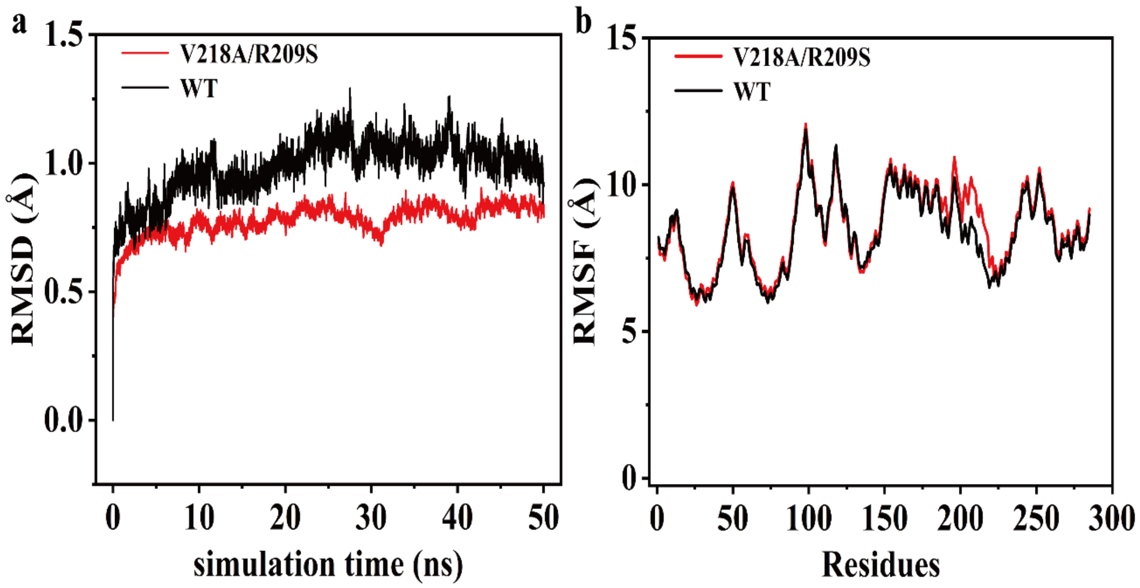

Figure S4. RMSD and RMSF of *Bm*Tyr^{WT} and *Bm*Tyr$^{V218A/R209S}$. (A) RMSD values calculated from MD simulations of *Bm*Tyr^{WT} and *Bm*Tyr$^{V218A/R209S}$; (B) RMSF values calculated from MD simulations of *Bm*Tyr^{WT} and *Bm*Tyr$^{V218A/R209S}$.

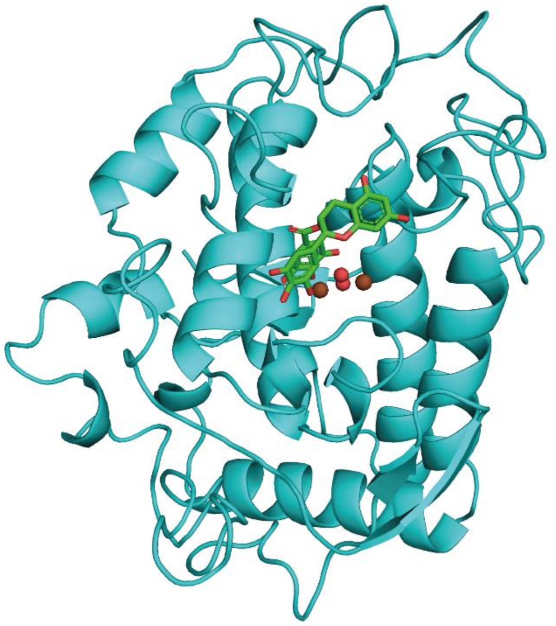

Figure S5. The whole TS2 state model.

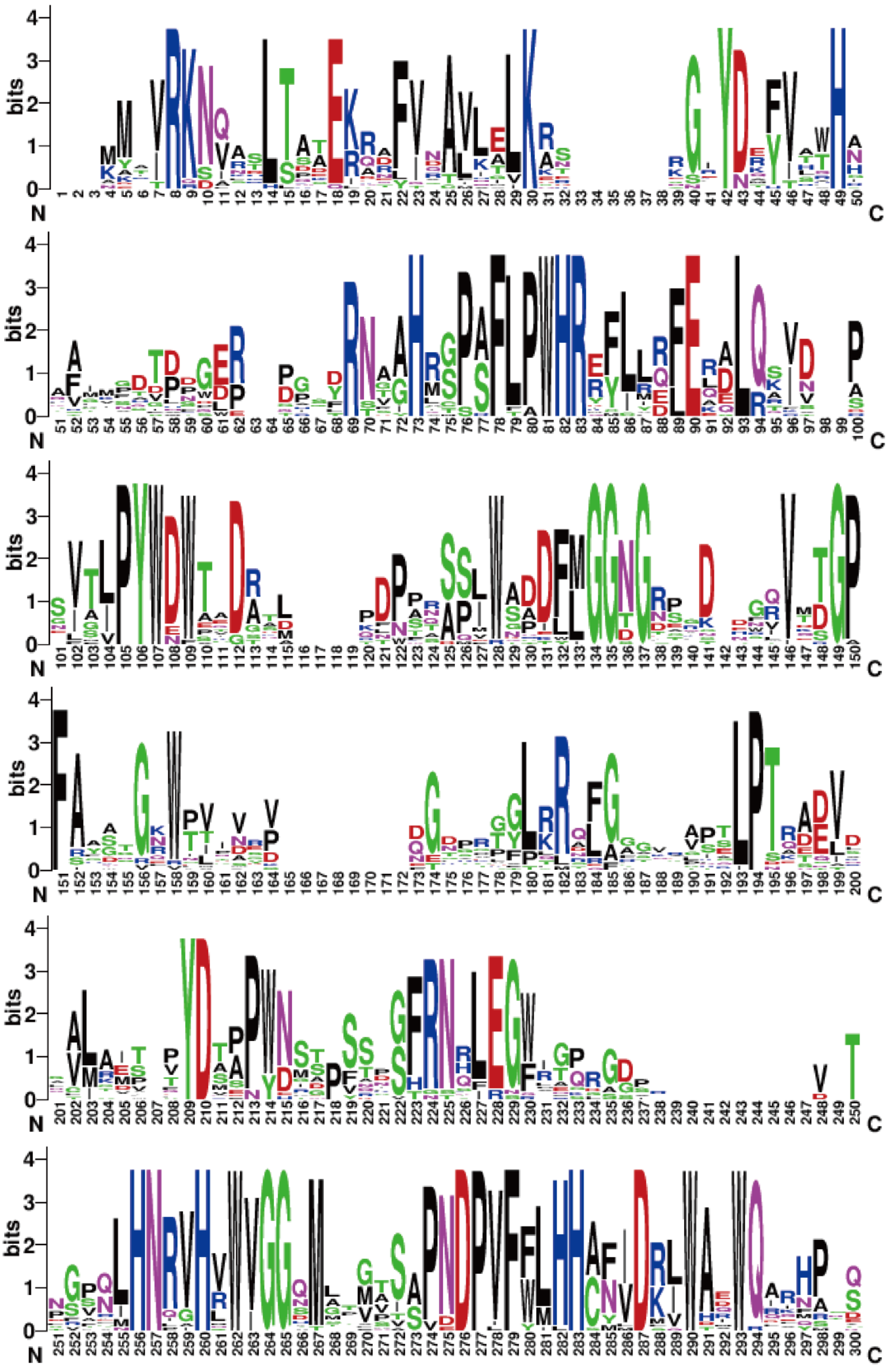

Figure S6. Multiple sequence alignment of *Bm*Tyr with homologous tyrosinase sequences from superfamily.

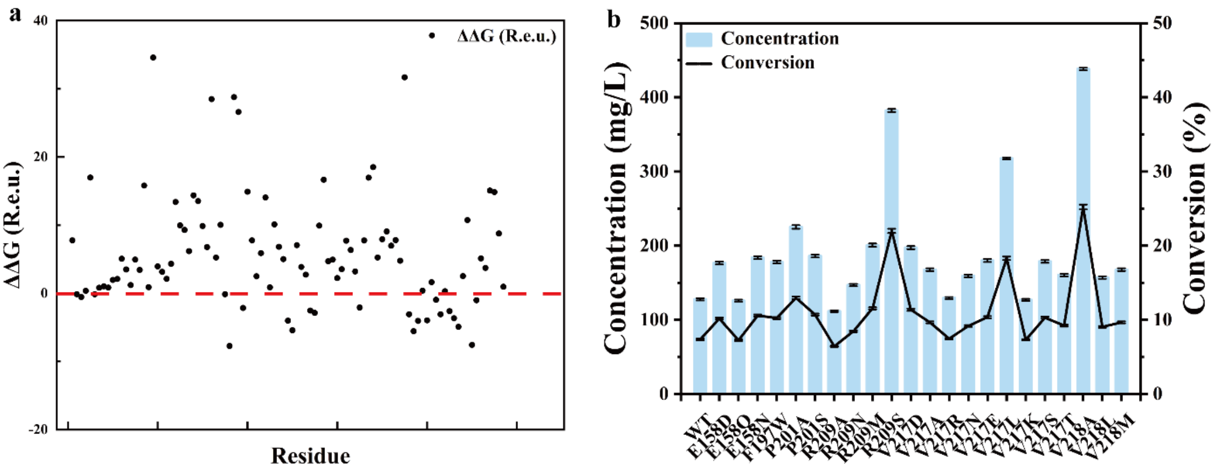

Figure S7. Redesign of *Bm*Tyr. (A) The energy difference between the wild type and the single point mutant was calculated (ΔΔG). (B) The activity of the designed enzymes on the TFDG production (5 mM substrates in Mcilvaine Buffer (0.2 M, pH 5.0), 30 °C, 0.1 mM Cu^{2+}). The data represent mean ± s.d., as determined from three independent experiments.

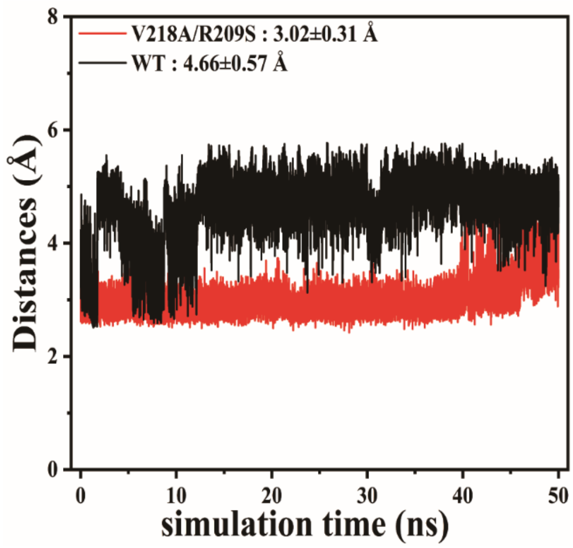

Figure S8. The distance between the -O11H of the hydroxy oxygen atom of EGCG and the O1 of the bridging oxygen atom of active cente.

Supplementary Material and Methods

Genetic constructions

The expression plasmid pET28a were purchased from Novagen (Madison, WI, U.S.A.). The host strain E coli BL21(DE3) was purchased from Invitrogen (Carlsbad, CA, U.S.A.). All genetic constructions were carried out by using standard molecular biology techniques. All chemicals and reagents were pure and obtained commercially. Molecular biology reagents such as the restriction enzymes, PrimeSTAR, DNA polymerase and polymerase chain reactions (PCR) reagents were supplied by TaKaRa (Dalian, China). The cloning inserts were created via PCR of ORFs of interest from their respective genomic or synthesis by GENEWIZ (Suzhou, China). DNA and protein sequence. The *Ec*1 was synthesized and codon optimized for *E. coli*. The optimized DNA sequence is as follow:

ATGAGTAAGCTGATTGTCCCGCAGTGGCCGCAGCCAAAAGGTGTTGCGGCCTGTAGCTC

CACTCGTATCGGCGGCGTGAGCTTGCCCCCGTATGACTCACTCAACCTCGGTGCCCATTG

TGGCGATAACCCGGATCACGTTGAGGAGAATCGCAAGCGACTTTTTGCTGCGGGCAATTT

GCCTTCTAAACCGGTCTGGCTTGAGCAGGTACACGGCAAAGATGTGCTTAAGCTCACTGG

CGAACCTTATGCCTCAAAACGGGCGGATGCCTCTTATAGCAATACGCCCGGCACGGTTTG

CGCAGTGATGACTGCCGACTGCCTCCCTGTGCTGTTTTGCAATCGAGCGGGAACGGAAG

TCGCCGCCGCTCATGCTGGCTGGCGTGGACTGTGCGCAGGCGTGCTGGAAGAGACGGTT

TCCTGTTTTGCTGATAATCCGGAAAATATTCTCGCCTGGTTAGGGCCGGCAATTGGTCCAC

GCGCGTTCGAAGTGGGGGGGGAGGTTCGCGAGGCGTTTATGGCAGTAGACGCTAAAGCA

AGTGCAGCTTTCATTCAGCATGGTGATAAGTATCTGGCGGATATTTATCAGCTTGCCCGGC

AGCGTCTGGCGAACGTGGGTGTTGAGCAAATTTTCGGCGGCGACCGTTGTACATATACGG

AAAATGAGACTTTCTTCTCTTATCGTCGCGACAAGACCACCGGTCGTATGGCAAGTTTCA

TTTGGCTGATATAA

Protein sequence:

MSKLIVPQWPQPKGVAACSSTRIGGVSLPPYDSLNLGAHCGDNPDHVEENRKRLFAAGNLPS

KPVWLEQVHGKDVLKLTGEPYASKRADASYSNTPGTVCAVMTADCLPVLFCNRAGTEVAA

AHAGWRGLCAGVLEETVSCFADNPENILAWLGPAIGPRAFEVGGEVREAFMAVDAKASAAF

IQHGDKYLADIYQLARQRLANVGVEQIFGGDRCTYTENETFFSYRRDKTTGRMASFIWLI

The *Ec*2 was synthesized and codon optimized for *E. coli*. The optimized DNA sequence is as follow:

ATGAGTAAGCTGATTGTCCCGCAGTGGCCGCTGCCAAAGGGTGTTGCGGCTTGTAGCTCT

ACTCGTATCGGCGGCGTGAGCTTGCCCCCGTATGACTCGCTCAACCTCGGTGCCCATTGT

GGCGATAACCCGGATCACGTTGAGGAGAATCGCAAGCGACTTTTTGCTGCGGGCAATTTG

CCTTCTAAACCGGTCTGGCTTGAGCAGGTACACGGCAAAGATGTGCTTAAGCTTACTGGC

GAACCTTATGCCTCAAAACGAGCAGATGCCTCTTATAGCAATACGCCTGGCACGGTTTGC

GCAGTGATGACTGCCGACTGCCTCCCTGTGCTGTTTTGCAATCGAGCGGGAACGGAAGT

CGCCGCCGCTCATGCTGGCTGGCGTGGACTGTGCGCAGGCGTGCTGGAAGAGACAGTTT

CCTGTTTTGCTGATAATCCGGAAAATATTCTCGCCTGGTTAGGGCCGGCAATTGGTCCACG

CGCGTTCGAAGTGGGGGCGGAGGTTCGCGAGGCGTTTATGGCAGCAGACGCCAAAGCG

AGTACAGCTTTCATTCAGCATGGTGATAAGTATCTGGCGGATATTTATCTGCTTGCCCGGC

AGCGTCTGGCGAGCGTTGGTGTTGAACAAATTTTCGGCGGCGACCGTTGTACATATACGG

AAAATGAGACTTTCTTCTCTTATCGTCGCGACAAGACCACCGGGCGTATGGCAAGTTTCA

TTTGGCTGATATAA

Protein sequence:

MSKLIVPQWPLPKGVAACSSTRIGGVSLPPYDSLNLGAHCGDNPDHVEENRKRLFAAGNLPS

KPVWLEQVHGKDVLKLTGEPYASKRADASYSNTPGTVCAVMTADCLPVLFCNRAGTEVAA

AHAGWRGLCAGVLEETVSCFADNPENILAWLGPAIGPRAFEVGAEVREAFMAADAKASTAF

IQHGDKYLADIYLLARQRLASVGVEQIFGGDRCTYTENETFFSYRRDKTTGRMASFIWLI

The *Bm* was synthesized and codon optimized for *E. coli.* The optimized DNA sequence is as follow:

ATGAGCAACAAATATCGCGTGCGCAAAAACGTGCTGCATCTGACCGATACCGAAAAACG

CGATTTTGTGCGCACCGTGCTGATTCTGAAAGAAAAAGGCATTTATGATCGCTATATTGCG

TGGCATGGCGCGGCGGGCAAATTTCATACCCCGCCGGGCAGCGATCGCAACGCGGCGCA

TATGAGCAGCGCGTTTCTGCCGTGGCATCGCGAATATCTGCTGCGCTTTGAACGCGATCTG

CAGAGCATTAACCCGGAAGTGACCCTGCCGTATTGGGAATGGGAAACCGATGCGCAGAT

GCAAGATCCGAGTCAGAGTCAGATTTGGAGCGCGGATTTTATGGGCGGCAACGGCAACC

CGATTAAAGATTTTATTGTGGATACCGGCCCGTTTGCGGCGGGCCGCTGGACCACCATTG

ATGAACAAGGCAACCCGAGCGGCGGCCTGAAACGCAACTTTGGCGCGACCAAAGAAGC

GCCGACCCTGCCGACCCGCGATGATGTGCTGAACGCGCTGAAAATTACGCAGTATGATAC

CCCGCCGTGGGATATGACGAGTCAGAACAGCTTTCGCAATCAGCTGGAAGGCTTTATTAA

CGGCCCGCAGCTGCATAACCGCGTGCATCGCTGGGTGGGCGGTCAGATGGGCGTGGTGC

CGACCGCGCCGAACGATCCGGTGTTTTTCTGCATCATGCGAACGTGGATCGCATTTGGG

CGGTGTGGCAGATTATTCATCGCAATCAGAACTATCAGCCGATGAAAAACGGCCCGTTTG

GTCAGAACTTTCGCGATCCGATGTATCCGTGGAACACCACCCCGGAAGATGTGATGAACC

ATCGCAAACTGGGCTATGTGTATGATATTGAACTGCGCAAAAGCAAACGCAGCAGCTAA

Protein sequence:

MSNKYKVRKNVLHLTDTEKRDFIRAVLILKEKGIYDRYIAWHGAAGKFHTPPGSDRNAAHM

SSAFLPWHREYLLRFERDLQSINPEVTLPYWEWETDAQMQDPSQSQIWSADFMGGNGNPK

KDFIVDTGPFAAGRWTTIDEQGNPSGGLKRNFGATKEAPTLPTRDDVLNALKITQYDTPPWD
MTSQNSFRNQLEGFINGPQLHNRVHRWVGGQMGVVPTAPNDPVFFLHHANVDRIWAVWQI
VHRNQNYQPMKNGPFGQNFRDSMYPWDTTPKDVMDHRKLGYVVYDIELRKSKRSS

The *An*1 was synthesized and codon optimized for *E. coli*. The optimized DNA sequence is as follow:

ATGACAATCTTTTTGCTACTCCTTGGGCTTCTAGCCCCACTGGTCCTCTGCAGTCCAATCA
GACACGTTACTCCGCATCTTTCCCACTCTACCCCAATACCGAGAGATATGCAAGGCAATTC
TTCGCAGTCACCCAATACCCCATGGCAGGGATACGATATAAACACGAACTACTATGAAAC
CATCCCACAGACCAATGTTGTCCGCGAATACTGGTTTGATATCGTCAACACGACCGCTGC
TCTTGATGGAGTTGAGAGACCAGTGCTTCTGGTCAACGGCCAGTTTCCAGGACCTACTAT
TGAGGCAAACTGGGGTGACACCGTTAAAGTTCATGTTACGAATCGCATGGAAAACAATG
GCACGGCCATCCACTTCCACGGAATTCGACAGCTCTACAACAACCAAATGGATGGCGTAG
CAGCGTTGACTCAGTGCCCTGTTCCTCCGAATTCCAGTTATACCTATGTCTGGCGAGCGGA
AGAATATGGGTCGAGTTGGTATCACTCACACTTCTCTCTCCAGGCATGGGAAGGGGTTTT
CGGAGGCATTTTGATTCACGGCCCTTCAACAGCGGAATATGACCACGACTTGGGAATGGT
CTTTCTCAATGACTGGTCGCATCAAACAGTCGACGAGATGTATCAATCTGTACTCGAGAG
TCAAAATCCGCCGCATTTTCAGACAGGCCTTATCAACGGAAGCAATATCTGGGTCACTGC
AGATAACCAAACCGTTGGTCGCAGATTCCAAACAGAGTTTGTACCAGGCCAGAGATACC
GGCTTCGGTTGGTCAATGCCGCCATGCATACCCATTTTAGGTTCTCAATCGATAATCATGA
CCTTACCGTTATTGCCAGTGATTTTGTTCCTATTGTACCATTCACGACGAACAACGTTCCC
ATCGGCATGGGACAACGGTATGATATTATCGTGACAGCAAACCAAGCTCCGGATAACTATT
GGATTCGTGCAATCCCACAGTCATTCTGCAGTGACAACGCGAATTCAGACAATATTAAAG
GAGTCCTCCATTACGAAGGTGCTGCCGATAACAGCGATCCCACGAGCACAAAGTGGGAT

TATGGTGACGACATTCAGTGCCTGGATTTCTCATTAGATGAGCTTGTCCCCTGGCTTGCCC

TTGATGCAGATATTGGCGGTGCCCAGATGGCCGAGTCAGACGTGGACTTCACTCCGTTCG

GGGATGTTCCTTTGTATTTGTGGACAATGGGAGGCAATGCCCTTAACATCTCGTGGAAAG

ATCCCACGTTACAGCAAACGTTCGAAGATCCCGACAAAATGGACTGGAAAGCAAGCCAG

GGCGTGATAGAGGCTGCGATCCCGAATAAATGGACCGTGTTGGTTGTCCAGACAGATCTT

CCTGTTCCTCACCCTATTCATTTGCATGGGCATGATTTCTATCTCCTTGCTCAGGGATTCGG

CCAATTCAATCCACAGAACGTCACGCTCAAAACGCATAACCCTCCCCGCCGCGACACTGC

GCTCATGACCGCTGCCACTCCTGAAAATGGTGGCGGCGGCTATATGGTTATTGGCTTCCCC

GCCGACAACCCGGGTGTATGGCTGATCCACTGCCACATTGGATTCCATGCAACAGAAGGA

TTCGCGCAGCAGATTGTCGAGCGACAGAGTGAGTTCAACACGTTCTTCAGCGAAGACCT

GCTGGAGAACACTTGTGATGCGTGGGATGAGTATGCCAAGGTGAATCCCTACGGACATCA

GTACCGGGCTCTCGCTGGGCCATACGAGTCGGGTATATAA

Protein sequence:

MTIFLLLLGLLAPLVLCSPIRHVTPHLSHSTPIPRDMQGNSSQSPNTPWQGYDINTNYYETIPQ

TNVVREYWFDIVNTTAALDGVERPVLLVNGQFPGPTIEANWGDTVKVHVTNRMENNGTAI

HFHGIRQLYNNQMDGVAALTQCPVPPNSSYTYVWRAEEYGSSWYHSHFSLQAWEGVFGGIL

IHGPSTAEYDHDLGMVFLNDWSHQTVDEMYQSVLESQNPPHFQTGLINGSNIWVTADNQTV

GRRFQTEFVPGQRYRLRLVNAAMHTHFRFSIDNHDLTVIASDFVPIVPFTTNNVPIGMGQRYD

IIVTANQAPDNYWIRAIPQSFCSDNANSDNIKGVLHYEGAADNSDPTSTKWDYGDDIQCLDF

SLDELVPWLALDADIGGAQMAESDVDFTPFGDVPLYLWTMGGNALNISWKDPTLQQTFEDP

DKMDWKASQGVIEAAIPNKWTVLVVQTDLPVPHPIHLHGHDFYLLAQGFGQFNPQNVTLK

THNPPRRDTALMTAATPENGGGGYMVIGFPADNPGVWLIHCHIGFHATEGFAQQIVERQSEF

NTFFSEDLLENTCDAWDEYAKVNPYGHQYRALAGPYESGI

The *An*2 was synthesized and codon optimized for *E. coli*. The optimized DNA sequence is as follow:

ATGCACTTGCATACTATCCTGGCTGCGGGCTGGCTCGCAGCCAGCGCCTATGCCGGGGTT

TCCTCTCGTTCCACGCAAGCATGCGCAGGAAACACACCGCACACCCGTGGCCAGTGGTG

TGATTTCGACATTCACACCGACTACTATGACGAAGCTCCGGACACTGGGGTAGTTCGCGA

ATATTGGTTGGAACTAGGAAACACCACCGCCGCTCCCGACGGCGTCCCTCGTAATGTCCT

AACCGTCAACGGGACGACTCCAGGGCCTACTCTATACGCCGATTGGGGTGACTGGGTCC

GAGTTCATGTATACAATGGTCTTGAAAACAATGGAACCAGCATCCATTGGCACGGTGTCC

GCCAGAACCACACCAACCCTCAAGACGGCACAAACTCCATTACCCAATGTCCCACGCCC

CCCGGAGGATCCGTCACGTACGAATGGCGCGCCACGCAGTACGGCACAACATACTACCA

CTCCCACTTTGCTCTGCAAGCATGGGAGGGCGTCTACGGTGGTATTGTGATCAACGGACC

TGCCAGCTCGAATTACGACGTCGACGCTGGCGTGCTTTATCTCATGGACTGGTCGCACCG

CACAGCCGACCAGATGTATTCCACCGCTGAAACGGACGGTGAACCAGAGATGACCACGG

GTTTGATTAATGGAACGAACATCTGGGTTAAGGAGAACAACGAAACGGTCGGTTCGCGC

TTCCTTATGAATGTGACTGCTGGCCAGTCTTACCGTCTTCGACTTATCAATGGTGGAATGA

GCAACTTGTTCCGATTCATGATCGATGATCATACGTTGACGGTTATTGCTGCGGACTTTGT

CCCCATCGAGCCTTACAATACGACTTCGCTTAATATTGGTGTTGGACAACGGTACGATGTG

ATCGTCACGGCAAACCAAGCCCAGGCTGGGTCTGACTTCTGGATGCGTGCTATCCCCCAG

GAGTCCTGTGCCGAGATTCAAAACACCGACATCAAGGGAATTTTCCATTATGAGCAGCCT

GACACTGCTGTAGCGACGCCCACAACAACAGCATACAACTATACCGACAGCTGCTACGA

CGAGCCATTGAGTAGCTTGGTGCCGGTAGTTGCCATCGACGCGTCTGGCGTCGGCTACAA

AAGTCATAACGAGGCGAACGTCATCATGAACAGTGCAGGCCTGTACAAGTGGTACATGG

GCCCGACCACGTTCAAGGCAGAATGGGACAACCCGACTTTGCTCCAGATCGCTAACGGC
AACACTTCATGGACTACACATCGCACGTTGTTGAAGTTGAAGGAAACGGCAAGTGGGTG
ATTGTTGACATTGAAATGGAAATCAATGTTCCTCATCCTATTCATCTCCACGGCCACGACT
TCTTCATCCTCGCCCAAGGCAAGGGAAGCTACACGTCGAACACGTCTCTGAACACTTAC
AACCCACCCCGTCGGGACACGGCCATGCTCCCTGCCCTCGGATATCTGGTCATCGCATTTA
AGACCGACAACCCTGGCGCGTGGCTTCTGCATTGCCATATTGGATGGCACCAATCGGAGG
GCTTCGCCATGCAGTTCGTGGAGAGCATCTCTCAGCTAGAGCCTATGATTGATGCTTCCGC
ACTCGAAAAGAACTGTGCTGCCTGGGATAAATATGCTGCTGCTAACCGCATTGAGGAGGA
TGATTCTGGTATCTAA

Protein sequence:

MHLHTILAAGWLAASAYAGVSSRSTQACAGNTPHTRGQWCDFDIHTDYYDEAPDTGVVRE
YWLELGNTTAAPDGVPRNVLTVNGTTPGPTLYADWGDWVRVHVYNGLENNGTSIHWHGV
RQNHTNPQDGTNSITQCPTPPGGSVTYEWRATQYGTTYYHSHFALQAWEGVYGGIVINGPA
SSNYDVDAGVLYLMDWSHRTADQMYSTAETDGEPEMTTGLINGTNIWVKENNETVGSRFL
MNVTAGQSYRLRLINGGMSNLFRFMIDDHTLTVIAADFVPIEPYNTTSLNIGVGQRYDVIVTA
NQAQAGSDFWMRAIPQESCAEIQNTDIKGIFHYEQPDTAVATPTTTAYNYTDSCYDEPLSSLV
PVVAIDASGVGYKSHNEANVIMNSAGLYKWYMGPTTFKAEWDNPTLLQIANGNTSWTNTS
HVVEVEGNGKWVIVDIEMEINVPHPIHLHGHDFFILAQGKGSYTSNTSLNTYNPPRRDTAML
PALGYLVIAFKTDNPGAWLLHCHIGWHQSEGFAMQFVESISQLEPMIDASALEKNCAAWDK
YAAANRIEEDDSGI

The *Bs*1 was synthesized and codon optimized for *E. coli*. The optimized DNA sequence is as follow:

ATGACACTTGAAAAATTTGTGGATGCTCTCCCAATCCCAGATACACTAAAGCCAGTACAG

CAATCAAAAGAAAAAACATACTACGAAGTCACCATGGAGGAATGCACTCATCAGCTCCAT

CGCGATCTCCCTCCAACCCGCCTGTGGGGCTACAACGGCTTATTTCCGGGACTGACCATT

GAGGTTAAAAGAAATGAAAACGTATATGTAAAATGGATGAATAACCTTCCTTCCACGCATT

TCCTTCCGATTGATCACACCATTCATCACAGTGACAGCCAGCATGAAGAGCCCGAGGTAA

AGACTGTTGTTCATTTACACGGCGGCGTCACGCCAGATGATAGTGACGGGTATCCGGAGG

CTTGGTTTTCCAAAGACTTTGAACAAACAGGACCTTATTTCAAAAGAGAGGTTTTTCATT

ATCCAAACCAGCAGCGCGGGGCTATATTGTGGTATCACGATCGCGCCATGGCGCTCACCA

GGCTAAATGTCTATGCCGGACTTGTCGGTGCATATATCATTCATGACCCAAAGGAAAAACG

CTTAAAACTGCCTTCAGACGAATACGATGTGCCGCTTCTTATCACAGACCGCACGACCAA

TGAGGATGGTTCTTTGTTTTATCCGAGCGCACCGGAAAACCCTTCTCCGTCACTGCCTAAT

CCTTCAATCGTTCCGGCTTTTTGCGGAGAAACCATACTCGTCAACGGGAAGGTATGGCCA

TACTTGGAAGTCGAGCCAAGGAAATACCGATTCCGTGTCATCAACGCCTCCAATACAAGA

ACCTATAACCTGTCACTCGATAATGGCGGAGATTTTATTCAGATTGGTTCAGATGGAGGGC

TCCTGCCGCGATCTGTTAAACTGAATTCTTTCAGCCTTGCGCCTGCTGAACGTTACGATAT

CATCATTGACTTCACAGCATATGAAGGAGAATCGATCATTTTGGCAAACAGCGCGGGCTG

CGGCGGTGACGTCAATCCTGAAACAGATGCGAATATCATGCAATTCAGAGTCACAAAACC

ATTGGCACAAAAAGACGAAAGCAGAAAGCCGAAGTACCTCGCCTCATACCCTTCGGTAC

AGCATGAAAGAATACAAAACATCAGAACGTTAAAACTGGCAGGCACCCAGGACGAATAC

GGCAGACCCGTCCTTCTGCTTAATAACAAACGCTGGCACGATCCCGTCACAGAAACACC

AAAAGTCGGCACAACTGAAATATGGTCCATTATCAACCCGACACGCGGAACACATCCGAT

CCACCTGCATCTAGTCTCCTTCCGTGTATTAGACCGGCGGCCGTTTGATATCGCCCGTTATC

AAGAAAGCGGGGAATTGTCCTATACCGGTCCGGCCGTCCCGCCGCCGCCAAGTGAAAAG

GGCTGGAAAGACACCATTCAAGCGCATGCAGGTGAAGTCCTGAGAATCGCGGCGACATT

CGGTCCGTACAGCGGACGATACGTATGGCATTGCCATATTCTAGAGCATGAAGACTATGAC

ATGATGAGACCGATGGATATAACTGATCCCCATAAATAA

Protein sequence:

MTLEKFVDALPIPDTLKPVQQSKEKTYYEVTMEECTHQLHRDLPPTRLWGYNGLFPGLTIEV

KRNENVYVKWMNNLPSTHFLPIDHTIHHSDSQHEEPEVKTVVHLHGGVTPDDSDGYPEAWF

SKDFEQTGPYFKREVFHYPNQQRGAILWYHDRAMALTRLNVYAGLVGAYIIHDPKEKRLKL

PSDEYDVPLLITDRTTNEDGSLFYPSAPENPSPSLPNPSIVPAFCGETILVNGKVWPYLEVEPR

KYRFRVINASNTRTYNLSLDNGGDFIQIGSDGGLLPRSVKLNSFSLAPAERYDIIIDFTAYEGES

IILANSAGCGGDVNPETDANIMQFRVTKPLAQKDESRKPKYLASYPSVQHERIQNIRTLKLAG

TQDEYGRPVLLLNNKRWHDPVTETPKVGTTEIWSIINPTRGTHPIHLHLVSFRVLDRRPFDIA

RYQESGELSYTGPAVPPPPSEKGWKDTIQAHAGEVLRIAATFGPYSGRYVWHCHILEHEDYD

MMRPMDITDPHK

The *Bs*2 was synthesized and codon optimized for *E. coli*. The optimized DNA sequence is as follow:

ATGAATACATATCACCCATTCAGTCTTACCACACCCTCGACACTCATGATACAAGACTGGG

CTCAAACGAATCAAAACAACAAAGAGGTCATTGCCGGATTTACGACAAAAAACGGCGGT

GTCAGCCAAAAGCCTTTTGAATCGTTAAATACAGGATTGCACGTTCATGACAAAGATGCA

GATGTAGTTAAAAATCGTGAATATATTGCCGATATGTTTAATACTGATTTGCAGTCTTGGGT

ATTCGCTGATCAGACACATGATAATCGCGTTCAGAAAGTGACGCAGAGGGATAGGGGAA

AAGGCGCCCGTGAGTATCACACGGCTCTAAAAGCAACGGACGGGCTCTATACAAATGAA

AAAAATGTATTTTTAGCATTATGCTTTGCTGATTGTGTGCCTCTTTTCTTTTATGATCCGGTT

AAGTCGCTTGTCGGAGTCGCCCATGCCGGGTGGAAAGGCACCGTCAAACAGATTGGCAG

AGAAATGGTGAAGCAATGGACTGAGAAGGAAGGTTCAAATCTCTCAGATATTTACGCTGT

TATTGGCCCGTCTATCAGCGGAGCATGCTATACGGTAGACGACCGCGTCATGGATGCTGTT

CGTGCATTGCCGGTTTCAGCAGACCTTGCCGCCAATCAGACGGCAAAGGCACAATATCAG

CTTGATCTGAAAGAGCTGAACCGTCTTATACTGATGGACAGCGGTTTGGCAAGTGAACAA

ATTTCTGTCAGCGGTTTATGCACGGAAAGCGAGCCGTCTCTTTTCTATTCTCATCGCCGCG

ATCAGGGGAAAACTGGACGGATGATGTCCTTTATCGGAATGAAGGAGGCATAA

Protein sequence:

MNTYHPFSLTTPSTLMIQDWAQTNQNNKEVIAGFTTKNGGVSQKPFESLNTGLHVHDKDAD

VVKNREYIADMFNTDLQSWVFADQTHDNRVQKVTQRDRGKGAREYHTALKATDGLYTNE

KNVFLALCFADCVPLFFYDPVKSLVGVAHAGWKGTVKQIGREMVKQWTEKEGSNLSDIYAV

IGPSISGACYTVDDRVMDAVRALPVSADLAANQTAKAQYQLDLKELNRLILMDSGLASEQIS

VSGLCTESEPSLFYSHRRDQGKTGRMMSFIGMKEA
